# Association between Low Serum Magnesium Level and Major Adverse Cardiac Events in Patients Treated with Drug-Eluting Stents for Acute Myocardial Infarction

**DOI:** 10.1371/journal.pone.0098971

**Published:** 2014-06-05

**Authors:** Guipeng An, Zhongqi Du, Xiao Meng, Tao Guo, Rui Shang, Jifu Li, Fengshuang An, Wenjing Li, Cheng Zhang

**Affiliations:** 1 The Key Laboratory of Cardiovascular Remodeling and Function Research, Chinese Ministry of Education and Chinese Ministry of Health, Shandong University Qilu Hospital, Jinan, Shandong, China; 2 Department of cardiology, Shandong University Qilu Hospital, Jinan, Shandong, China; 3 Fine Arts School of Shandong University, Jinan, Shandong, China; University of Louisville, United States of America

## Abstract

**Objectives:**

We investigated the association of serum magnesium (Mg) levels and major adverse cardiac events (MACEs) after drug-eluting stent (DES) implantation.

**Background:**

Mg depletion plays a key role in the pathphysiologic features of diabetes mellitus, hypertension, thrombosis, arrhythmias and coronary artery disease. Whether the depletion is related to the long-term prognosis of DES implantation is not known.

**Methods:**

*From 2008 to 2011, we enrolled 414 consecutive patients <50 years old* who underwent DES implantation for acute coronary syndrome. Serum Mg level was analyzed and patients were followed up for a median of 24 months (interquartile range 14–32 months) for the occurrence of MACEs defined as death, myocardial infarction, stroke, and any revascularization.

**Results:**

For patients with unstable angina, no significant association between serum Mg level and MACEs was found in the multivariate model. For patients with myocardial infarction, after adjusting for age, positive family history, smoking status, hypertension, hypercholesterolemia, and diabetes at baseline, the risk was 8.11-fold higher for patients with quartile 1 than 4 Mg level (95% confidence interval 1.7–38.75; P<0.01). In addition, when tested as a continuous variable, serum magnesium was a significant predictor for MACEs of acute myocardial infarction (HR [per 0.1 mM increase], 0.35 [95% CI, 0.19–0.63], p< 0.01), after adjustment for other confounders.

**Conclusions:**

Low serum level of Mg may be an important predictor of MACEs with DES implantation for acute myocardial infarction. Further research into the effectiveness of Mg supplementation for these patients is warranted.

## Introduction

Percutaneous coronary intervention (PCI) with drug-eluting stents (DESs) has been used widely to treat coronary heart disease, with relatively reduced restenosis and target lesion revascularization rates as compared with bare-metal stents [1], [2]. However, restenosis is not completely diminished and has increased in absolute number because of the increasing number of implanted DESs as well as treatment of more complex lesions [3]. In addition, stent thrombosis remains a unique severe complication after DES implantation because of high morbidity and mortality [4]. How to identify and manage patients at high risk for these complications has become an emerging issue.

The important human element magnesium (Mg) is an activator of more than 300 enzymes. Thus, Mg plays an important role in numerous diverse diseases including cardiovascular disorders [5]. Several studies have indicated the relationship between Mg and the prognosis of coronary artery disease (CAD). Data from the National Health and Nutrition Examination Survey Epidemiologic Follow-up Study (NHANES) showed that serum Mg level was inversely associated with cardiovascular related deaths and hospitalizations [6], [7]. In a northern German population-based sample, a low serum Mg level was a significant independent predictor of all-cause and cardiovascular mortality after adjustment for cardiovascular risk factors including diabetes and hypertension [8].

However, the relationship between serum Mg level and the prognosis with DES implantation is not clearly understood. Hypomagnesemia was found associated with poor glycemic control and various long-term complications of diabetes mellitus, an important risk factor for in-stent restenosis and stent thrombosis[9]–[11]. Mg deficiency was found able to enhance vascular endothelial injury and promote platelet-dependent thrombosis, for possible involvement in stent thrombosis [12], [13]. As well, a beneficial trend of Mg treatment preventing acute recoil and late restenosis (within 6 months) was found after percutaneous transluminal coronary angioplasty [14].

Here, we investigated the association of serum Mg level and a combined endpoint of death, myocardial infarction, stroke, and any revascularization (major adverse cardiac events [MACEs]) in patients receiving DES implantation for acute coronary syndrome (ACS).

## Subjects and Methods

### Protocols

The study conformed to the guiding principles of the Declaration of Helsinki and was approved by the local review board and ethics committee of Shandong University. All patients gave their written informed consent to participate.

The study was a prospective cohort study. We included consecutive patients <50 years old who underwent DES implantation for ACS from January 2008 to December 2011 in Qilu Hospital, Shandong University. Exclusion criteria were no serum Mg record, history of familial dyslipidemia, type I diabetes, endstage renal disease or receiving any Mg supplementation. We listed history of familial dyslipidemia as one of the exclusion criteria because of its probable confounding effect. All patients came from the same geographical area and had a similar socioeconomic and ethnic background.

### Patient Data

At admission, 2 independent observers collected data on medical history, physical examination, results of laboratory examination and extent of CAD. Clinical history taking and physical examination paid special attention to cardiovascular risk factors and comorbidities: age, sex, smoking, drinking, family history of CAD, hypertension, diabetes mellitus, hypercholesterolemia, renal failure, heart failure, history of using diuretic drugs, previous acute myocardial infarction, previous coronary artery bypass grafting, and previous PCI. In case of discrepancies, both investigators reevaluated the data to achieve consensus.

A positive family history was defined as the presence of at least one-first degree relative with CAD before age 55 years for men and age 65 years for women. Body mass index (BMI) was calculated as weight (kg) divided by height (m) squared. Prevalent diabetes was defined as a fasting serum glucose >126 mg/dl or current use of any diabetes medication. Prevalent hypertension was defined as seated diastolic blood pressure >90 mmHg, systolic blood pressure >140 mmHg, or use of anti-hypertensive drugs. Hypercholesterolemia was defined as total cholesterol level >5.2 mmol/L. Current smokers or drinkers were defined as self-reported regular smoking or drinking.

We obtained antecubital venous blood samples for determining serum Mg level at admission for patients with unstable angina or 48 to 72 hr later after the onset of myocardial infarction as described [15], [16]. Serum Mg concentration was determined on a Roche COBAS 8000 analyzer with the use of the xylidyl blue reaction according to the manufacturer’s instructions. The reference value is 0.65 to 1.1 mmol/L, and the intra-test coefficient of variation is 1.2%.

Coronary angiography involved the Judkin technique with a quantitative coronary angiographic system. CAD extent was assessed as previously described [17]: The number of major coronary vessels with >70% occlusion (left anterior descending, left circumflex, and right coronary arteries), namely, single-, double-, or triple-vessel disease, was classified as grades 1 to 3; >50% lesion in the left main coronary artery was considered two-vessel disease. Coronary angiograms were interpreted by 2 investigators who were blinded to patient data.

### Study Endpoints and Definitions

Follow-up information for patients in terms of health status and hospital admissions was obtained from hospital records and by telephone in June 2012. The primary endpoint was the occurrence of MACEs including death (noncardiacvascular death and cardiac death), myocardial infarction, stroke, and any revascularization (PCI or coronary artery bypass grafting). Cardiovascular death was defined as death from myocardial infarction, stroke or sudden death without any obvious reasons. The definition of myocardial infarction was based on the third universal definition [18]. Target lesion revascularization was defined as any attempted percutaneous or surgical revascularization of lesions in the coronary artery after the initial procedure. Outcomes were assessed by 2 independent observers who were blinded to patient baseline, clinical, and laboratory data.

### Statistical Analysis

Analysis of normality of continuous variables involved the Kolmogorov-Smirnov test. Data for normally distributed continuous variables are expressed as mean±SD. Otherwise, data are presented as median (interquartile range [IQR]). Categorical data are expressed as number and percentage. Differences were analyzed by ANOVA, Kruskal-Wallis test, or chi-square test as appropriate. Event-free survival by baseline serum Mg level (in quartiles) was analyzed by Kaplan-Meier curves and compared by log rank test. Potential confounders associated with clinical outcome were examined by a Cox proportional hazards multiple regression model with p<0.2 for variable inclusion in the model. A two-sided p<0.05 was considered statistically significant. All analyses involved use of SPSS 17.0 for Windows (SPSS, Inc., Chicago, IL, USA).

## Results

We included 414 patients with acute coronary syndrome (median age 45 years [IQR 41–47 years]; 373 male [90.1%]). Median serum Mg concentration at admission was 0.92 mmol/L (0.87–0.97 mmol/L). For further analysis, patients were divided into 4 groups by serum Mg quartile: quartile 1, <0.87 mmol/L; quartile 2, 0.87 to 0.91 mmol/L; quartile 3, 0.92 to 0.96 mmol/L; and quartile 4, >0.96 mmol/L. The prevalence of baseline risk factors is in Table I. Most risk factors were almost equally balanced among serum Mg quartiles except for smoking and diabetes mellitus: high frequency of smoking (P = 0.001) and diabetes (P<0.05) was associated with low Mg level. However, Mg level was lower for patients with than without diabetes (P = 0.03) and smoking versus not smoking (P = 0.01) (data not shown).

**Table 1:** Risk factors of coronary artery disease by quartile (Q) of serum magnesium (Mg) level.

	Serum Mg level (mM)				
	Q1	Q2	Q3	Q4	*P* value
Serum Mg level(mM)	<0.87	0.87–0.91	0.92–0.96	>0.96	
No. of patients	100	95	107	112	
Age, years (no, range)	44 (40–47)	44 (40–47)	45 (41–47)	45 (42–47)	0.46
Men	91 (91)	90 (94.7)	92(86)	100(89.3)	0.21
Smoking	74 (74)	71 (74.7)	55(51.4)	75(67)	0.001
Drinking	67 (67)	61 (62.2)	55(51.4)	59(52.7)	0.45
BMI (kg/m^2^; no, range)	26.12 (23.98–29.39)	27.39 (24.68–29.05)	26.2 (23.56–29.3)	27.02 (24.59–29.31)	0.56
Family history of CAD	33 (33)	28 (29.5)	33(28)	32(28.6)	0.91
Hypertension	54 (54)	47 (49.5)	46(43)	50(44.6)	0.38
Diabetes mellitus	36 (36)	22 (23.2)	20(18.7)	26(23.2)	0.03
Hypercholesterolemia	28 (28)	26(27.4)	37(34.6)	40(35.7)	0.44

BMI, body mass index; CAD, coronary artery disease.

Data are no. (%) unless indicated.

The clinical characteristics of patients with ACS are in Table II. Clinical presentation differed by Mg level (P<0.01); increased frequency of myocardial infarction was associated with low Mg level and increased frequency of unstable angina with high Mg level, with no association of Mg level and left ventricular ejection fraction, extent of CAD, bifurcations, and stent number per patient. One patient with Mg level in quartile 4 had a previous diagnosis of renal failure; 2 patients with Mg level in quartiles 1 and 3 had a history of heart failure and using diuretics drugs. In all, 21 patients had a history of myocardial infarction; 6 had undergone PCI.

**Table 2:** Clinical characteristics of patients with acute coronary syndrome by quartiles of serum Mg level.

	Serum Mg level (mM)				
Characteristics	Q1	Q2	Q3	Q4	*P* value
Clinical presentation					
Unstable angina	54(54)	42 (44.2)	60 (56.1)	77 (68.8)	0.005
Myocardial infarction	46(46)	53 (55.8)	47 (43.9)	35 (31.2)	
Renal failure	0	0	0	1	
Chronic heart failure	1	0	1	0	
Use of diuretics drugs	1	0	1	0	
Previous acute myocardial infarction	6	3	7	5	
Previous coronary artery bypass grafting	0	0	0	0	
Previous percutaneous coronary intervention	1	1	2	2	
Left ventricular ejection fraction	0.57±0.13	0.58±0.09	0.59±0.12	0.61±0.09	0.35
Diseased coronary artery					
Left main	2(2)	1(1.1)	2(1.9)	2(1.8)	
Single vessel	43(43)	40(42.1)	52(48.6)	39(34.8)	0.23
Multi-vessels	57(57)	55(57.9)	55(51.4)	73(65.2)	
Bifurcations	15(15)	10(10.5)	10(9.3)	20(17.9)	0.23
Stent number per patient					
1–2	67(67)	67(70.5)	85(79.4)	74(66.1)	0.12
>3	33(33)	28(29.5)	22(20.6)	38(33.9)	

Data are no. (%) unless indicated.

During a median follow-up of 24 months (IQR 14–32 months), no patient was lost to follow-up. Overall, 2 patients died from cardiac causes and 1 patient from liver cancer (Table III), for an overall mortality of 0.7%. No stroke was recorded. A total of 17 patients experienced myocardial infarction; the incidence was 4.1%. Repeat PCI was required in 40 patients (9.7%) and coronary artery bypass grafting in 2 (0.5%). The incidence of all MACEs was 12.6%.

**Table 3:** Factors of major adverse cardiac events (MACEs) and relative risk by quartiles of serum Mg level.

	Serum Mg level (mM)			
	Q1	Q2	Q3	Q4
No. of MACEs	16	15	10	11
Death	1	2	0	0
Myocardial infarction	7	3	3	4
Cerebrovascular accident	0	0	0	0
Repeat PCI	11	11	9	9
Coronary artery bypass grafting	1	1	0	0
In-stent restenosis	7	6	5	5
Stent thrombosis	3	3	2	0
**Relative risk** (95% CI)				
Aged-adjusted model	2.63 (1.21–5.71)*	2.04 (0.92–4.5)	0.98 (0.41–2.31)	1
Multivariate model #	2.3 (1.05–5.03) *	1.93 (0.87–4.28)	0.94 (0.4–2.25)	1

PCI, percutaneous coronary intervention; 95% CI, 95% confidence interval.

*P<0.05.

# adjusted for positive family history, smoking status, hypertension, hypercholesterolemia, and diabetes at baseline.

**Figure 1:**
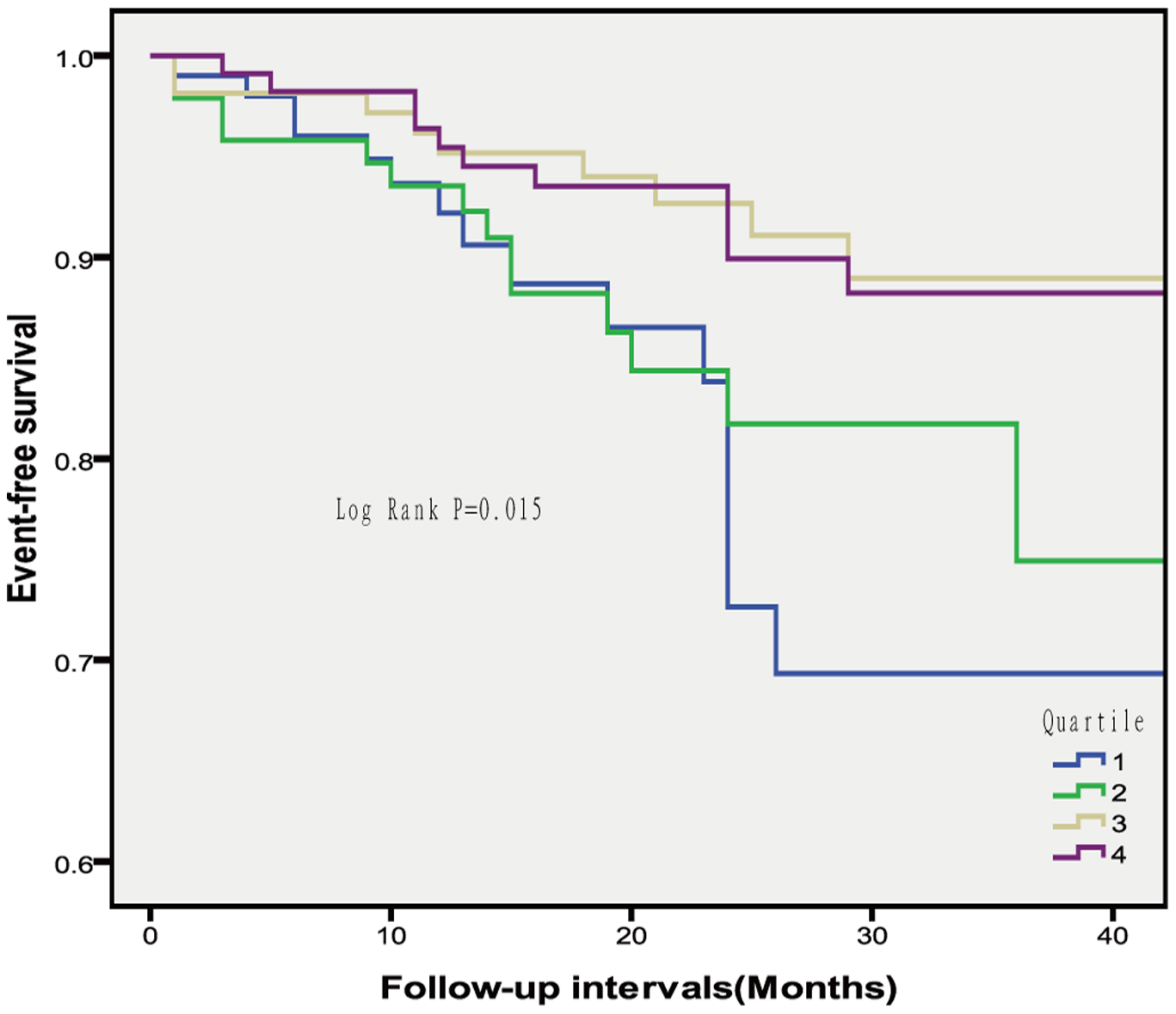
Cumulative major adverse cardiac event-free survival in 414 patients by quartiles of serum magnesium level.

MACE-free survival differed by Mg level (P = 0.015, Figure 1). The incidence of MACEs was higher for patients with quartile 1 than 4 Mg level (P = 0.009), whereas patients with quartiles 4 and 3 Mg levels showed comparable event-free survival. We assessed the association of Mg level and risk of MACEs by a multivariate Cox proportional hazards model (Table III). After adjusting for age, the risk was 2.63-fold higher for patients with quartile 1 than 4 Mg level (95% confidence interval 1.21–5.71; P = 0.014). After adjusting for positive family history, smoking status, hypertension, hypercholesterolemia, and diabetes at baseline, the association persisted, with a 2.3-fold increased risk for patients with quartile 1 versus 4 Mg level (1.05–5.03; P = 0.033).

To clear the probable confounding effect of different clinical presentations, the association between serum magnesium and MACEs after DES implantation was assessed by unstable angina and acute myocardial infarction separately. However, for patients with unstable angina, no significant association was found in the multivariate model (Table IV). For patients with myocardial infarction, after adjusting for age, positive family history, smoking status, hypertension, hypercholesterolemia, and diabetes at baseline, the risk was 8.11-fold higher for patients with quartile 1 than 4 Mg level (95% confidence interval 1.7–38.75; P<0.01, Table IV). In addition, Mg levels were tested as a continuous variable. Multivariate Cox proportional hazard analysis demonstrated that serum magnesium was a significant predictor for MACEs of acute myocardial infarction (HR [per 0.1 mM increase], 0.35 [95% CI, 0.19–0.63], p< 0.01), after adjustment for other confounders, such as age, positive family history, smoking status, hypertension, hypercholesterolemia, and diabetes at baseline.

**Table 4:** Relative risk by quartiles of serum Mg level according to different clinical presentation.

	Serum Mg level (mM)			
	Q1	Q2	Q3	Q4
**Unstable angina**				
Serum Mg level(mM)	<0.87	0.87–0.92	0.93–0.98	>0.98
No. of patients	54	59	60	60
No. of MACEs	8	9	6	9
Death	1	1	0	0
Myocardial infarction	2	0	1	3
Repeat PCI	5	7	6	7
Coronary artery bypass grafting	1	1	0	0
Relative risk (95% CI) #	1.38(0.51–3.72)	1.1 (0.42–2.9)	0.63(0.22–1.83)	1
**Myocardial infaction**				
Serum Mg level(mM)	<0.86	0.86–0.90	0.91–0.94	>0.94
No. of patients	41	46	47	47
No. of MACEs	8	7	3	2
Death	0	1	0	0
Myocardial infarction	5	3	1	2
Repeat PCI	6	5	3	1
Coronary artery bypass grafting	0	0	0	0
Relative risk (95% CI) #	8.11(1.7–38.75) **	6.47(1.3–32.2) *	2.05(0.34–12.33)	1

95% CI, 95% confidence interval.

*P<0.05, **P<0.01.

# adjusted for age, positive family history, smoking status, hypertension, hypercholesterolemia, and diabetes at baseline.

## Discussion

We found serum Mg levels, independent of other risk factors, is inversely related to the incidence of MACEs in patients with DES implantation for acute myocardial infarction but not unstable angina. Compared with patients with the highest serum Mg level (>0.94 mmol/L), those with the lowest level (<0.86 mmol/L) showed an 8.11-fold higher risk for MACEs after DES implantation. This finding can allow us to better distinguish patients at increased risk and support further research into the effectiveness of Mg supplementation for this group of patients.

Mg deficiency enhances vascular endothelial injury, increases low-density lipoprotein concentration and oxidative modification, and thus promotes the development and progression of atherosclerosis [19], [20]. As well, it affects risk factors of myocardial infarction such as blood pressure, glucose metabolism, and lipid levels [21], [22]. In addition, Mg has antiarrhythmic effects, and chronic Mg deficiency may be proarrhythmic [23]. Several recent studies showed an association of low serum Mg level and increased risk of atrial fibrillation [24] and sudden cardiac death [25]. Therefore, low Mg serum level may be related to the prognosis of DES implantation. With a median follow-up of 24 months for ACS patients, our data confirmed this hypothesis, finding an inverse relationship between serum Mg level and MACEs in patients with DES implantation for acute myocardial infarction.

Mg is the physiological Ca antagonist, and its serum concentration is remarkably constant in healthy subjects. Low Mg level deteriorates endothelial function [26] and leads to the Ca overload that occurs after reperfusion [27]. Mg was found involved in platelet-dependent thrombosis and inversely related to platelet aggregation and adenosine triphosphate release [28], [29]. In clinical experiments, Mg supplementation could reduce acute platelet-dependent thrombosis [28]. In addition, Mg can halt smooth muscle cell proliferation and stimulate endothelial cell proliferation, which might translate into a beneficial effect in the setting of stent-associated vascular injury [30]. These may be potential mechanisms for the decreased MACEs seen in our patients with low Mg levels. However, the reason why the relationship was found only in patients with acute myocardial infarction but not unstable angina still needs to be investigated.

Some limitations of the present study must be acknowledged. The main limitation is that most of the end points were repeated PCI. A much larger sample size would be needed to have necessary power for a more robust death/myocardial infarction endpoint. Secondly, the sample was from a single medical center, so results may not be representative of the general population. Furthermore, we investigated relatively young ACS patients. Our conclusions need to be revaluated before application to older patients.

## Conclusions

We found that low serum level of Mg predicts MACEs in patients with DES implantation for acute myocardial infarction but not unstable angina. Many questions remain to be addressed about the pathophysiologic mechanism of the effect of Mg level on the prognosis of DES implantation. Meanwhile, the efficiency of Mg treatment and the ability to administer Mg in a predictable manner after DES implantation for acute myocardial infarction need to be elucidated with randomized controlled trials.
